# Virtual surgical plan with custom surgical guide for orthognathic surgery: systematic review and meta-analysis

**DOI:** 10.1186/s40902-024-00449-2

**Published:** 2024-11-14

**Authors:** Yoon-Jo Lee, Ji-Hyeon Oh, Seong-Gon Kim

**Affiliations:** Department of Oral and Maxillofacial Surgery, College of Dentistry, Gangneung–Wonju National University, Gangneung, 25457 Republic of Korea

**Keywords:** Orthognathic surgery, Custom surgical guides, 3D printing, CAD/CAM Technology, Surgical planning, Patient-specific design

## Abstract

**Background:**

The shift from traditional two-dimensional (2D) planning to three-dimensional (3D) virtual surgical planning (VSP) has revolutionized orthognathic surgery, offering new levels of precision and control. VSP, combined with computer-aided design/computer-aided manufacturing (CAD/CAM) technology, enables the creation of patient-specific surgical guides and implants that translate preoperative plans into more precise surgical outcomes. This review examines the comparative accuracy and operative efficiency of VSP, especially when used with custom surgical guides, against conventional 2D planning in orthognathic surgery.

**Main text:**

The study systematically reviewed and analyzed published literature comparing the accuracy and operative time between VSP and conventional planning methods. The meta-analysis included clinical trials, controlled trials, and observational studies on patients undergoing orthognathic surgery, focusing on the degree of alignment between planned and postoperative bone positions and total surgery time. Results indicate that VSP consistently reduces discrepancies between planned and actual surgical outcomes, particularly when integrated with custom surgical guides. Additionally, while VSP demonstrated potential time-saving advantages over conventional planning, these differences were not statistically significant across studies, likely due to high variability among study protocols and designs.

**Conclusions:**

VSP with custom surgical guides enhances surgical accuracy in orthognathic procedures, marking a significant advancement over traditional methods. However, the reduction in operative time was not conclusively significant, underscoring the need for further studies to evaluate time efficiency. These findings emphasize VSP’s role in improving surgical precision, which holds substantial implications for future orthognathic surgical practices.

## Background

In the last decade, the transition from traditional two-dimensional (2D) methods to three-dimensional (3D) virtual surgical planning (VSP) has accelerated in orthognathic surgery [1]. Virtual surgical planning and 3D printing have significantly expanded the possibilities in oral and maxillofacial surgery, particularly for orthognathic procedures [2]. The integration of CAD/CAM surgical splints into orthognathic surgery enables precise simulation of jaw movements and accurate execution of surgical plans in the operating room [1, 3]. Additionally, 3D-printed personalized surgical guides and patient-specific implants (PSI) not only facilitate accurate osteotomies and ensure proper fitting of osteosynthesis plates but also introduce a groundbreaking waferless approach that diverges substantially from traditional wafer-guided jaw fixation techniques [2].

The advent of custom surgical guides represents a pivotal transition in orthognathic surgery, moving away from reliance on surgeon experience and manual techniques that often resulted in challenges in achieving precise and consistent outcomes [4, 5]. The introduction of these guides has greatly changed the process, making it possible to achieve a level of precision that was not attainable before [6].

Custom surgical guides are meticulously designed using patient-specific anatomical data derived from advanced imaging techniques. This personalized approach enhances both the accuracy and efficiency of surgical interventions [6]. By aligning surgical procedures closely with preoperative planning, these guides reduce operative time and improve overall surgical outcomes [5]. The critical advantage of custom surgical guides lies in their ability to translate complex preoperative planning into actionable, patient-specific steps [7]. This significantly improves the predictability of surgical results, making custom surgical guides indispensable tools in modern surgical protocols and marking a significant advancement in the field [6, 7].

The development of these guides has been propelled by advancements in technologies such as cone beam computed tomography (CBCT) and 3D printing [6]. CBCT provides highly accurate, three-dimensional imaging of craniofacial structures, enabling detailed anatomical analysis, which is crucial for customizing surgical guides [8]. Meanwhile, 3D printing technology has transformed the fabrication of these guides, allowing for the creation of intricate, patient-specific surgical tools with unprecedented precision [9]. The synergy between these technological advancements and medical practice has proven transformative, improving the accuracy and reliability of surgical guides while enhancing the effectiveness of orthognathic procedures [5]. This evolution reflects a remarkable integration of innovation and clinical application, highlighting the dynamic nature of modern medical practices [10].

This article aims to comprehensively compare the accuracy and operative time between 3D virtual surgical planning and traditional 2D conventional surgical planning. Furthermore, it seeks to evaluate the accuracy of virtual surgical planning that includes custom surgical guides in contrast to 2D conventional surgical planning. Through this analysis, the article will highlight the significant impact of these advancements on surgical precision and overall effectiveness in orthognathic surgery.

## Methods

This study constitutes a systematic review and meta-analysis. We formulated the research protocol following the guidelines provided by the Preferred Reporting Items for Systematic Reviews and Meta-Analysis Protocols (PRISMA-P). This meta-analysis has been registered with PROSPERO, registration number [CRD42024585756].

### PICOS

The search methodology followed the PICO framework, representing P (Patient Population), I (Intervention or Exposure for observational studies), C (Comparison), and O (Outcomes). In this systematic review, we applied the PICOS approach, covering the study population (in patients undergoing orthognathic surgery), interventions (Three-dimensional virtual surgical planning of orthognathic surgery), comparison (two-dimensional conventional surgical planning of orthognathic surgery), and outcomes of interest (the accuracy of operation, surgical time) (Table 1).

**Table 1:** The search methodology followed the PICO framework

P (patient population)	In patients undergoing orthognathic surgery
I (Intervention or Exposure for observational studies)	Three-dimensional virtual surgical planning of orthognathic surgery
C (Comparison)	Two-dimensional conventional surgical planning of orthognathic surgery
O (Outcomes)	The accuracy of operation and surgical time
S (Study design)	Clinical trials, controlled trials, retrospective and prospective studies, and case series
Focused question	Are there any differences in the accuracy of operation and surgical time between ‘Three-dimensional virtual surgical planning’ and ‘Two-dimensional surgical planning’?

### Eligibility criteria

Comparative studies (prospective or retrospective studies and randomized controlled trials) in humans assessing the accuracy of orthognathic surgical planning using VSP compared with conventional surgical planning (CSP) were included. The inclusion criteria for this study encompassed full-text articles involving patients who underwent orthognathic surgery, specifically those that utilized three-dimensional VSP. The primary outcomes assessed were the accuracy of the operation and surgical time. Eligible study designs included clinical trials, controlled trials, retrospective and prospective studies, and case series.

To ensure the reliability and relevance of the findings, several exclusion criteria were applied. Studies were excluded if they lacked essential data on accuracy in orthognathic surgery, as incomplete data could compromise the comprehensiveness of the analysis. Articles published in languages other than English were not considered, and non-peer-reviewed literature—such as conference abstracts, posters, and unpublished studies—was excluded to maintain the quality and rigor of the evidence.

Research involving non-human subjects, including animal models, surgical simulations, and rapid prototype models, was excluded because their outcomes might not directly translate to human patients undergoing orthognathic surgery. Studies focusing on populations not representative of typical orthognathic surgery patients—such as individuals with congenital craniofacial anomalies or trauma-related injuries—were also excluded. Additionally, studies concentrating solely on procedures like genioplasty or malarplasty were not included. Studies exclusively involving pediatric patients were excluded due to differing anatomical considerations, surgical techniques, and complication profiles compared to adult patients. Research focusing solely on patients with specific medical comorbidities, such as severe cardiovascular disease or uncontrolled diabetes, was omitted to maintain a homogeneous study population and reduce confounding factors that could affect complication rates.

To include the most current evidence, studies published before a specified date were not considered. Duplicate studies or redundant data from the same study cohort were excluded to avoid repetition and to ensure the integrity of the analysis. Lastly, articles lacking complete demographic information were not included, as such information is crucial for contextualizing the study results.

### Information sources

Following the guidelines outlined in the PRISMA statement, we conducted an electronic search of various databases, including PubMed and Scopus. The manual search also encompassed the bibliographies of all articles chosen for full-text screening, along with previously published reviews pertinent to this systematic review. We included studies published between 2014 and 2024 in our meta-analysis. Two reviewers (LYJ and KSG) performed the study selection independently. In the event of disagreement between the reviewers, the third reviewer (OJH) was consulted.

### Search strategy and article selection

A MEDLINE(PubMed) and Scopus search was conducted. The search only included articles published in English, from year 2014 until 2024. We used the Boolean operators ‘OR’ to broaden the search and ‘AND’ to combine different areas. The search equations for each database were as follows:

- PubMed: ((((Accuracy) OR (precision)) AND (Orthognathic surgery)) AND (time)) OR (((Accuracy) OR (precision)) AND (orthognathic surgery)) OR ((time) AND (orthognathic surgery)).
- Scopus: (TITLE-ABS-KEY(“Orthognathic surgery” AND “time”) OR TITLE-ABS-KEY((“Orthognathic surgery” AND (“Accuracy” OR “Precision”)) OR TITLE-ABS-KEY(“Orthognathic surgery” AND (“Accuracy OR Precision”) AND “time”)) AND PUBYEAR > 2013 AND PUBYEAR < 2025 AND ( LIMIT-TO (LANGUAGE, “English”)) AND (LIMIT-TO (SUBJAREA, “MEDI”) OR LIMIT-TO (SUBJAREA, “DENT”)) AND (LIMIT-TO (DOCTYPE, “ar”) OR LIMIT-TO (DOCTYPE, “cp”)).

Two independent reviewers, LYJ and KSG, evaluated the titles and abstracts of all studies found in the initial search. If the abstracts did not provide sufficient information, the reviewers examined the full text to determine whether to include or exclude the studies. The authors reviewed the full texts of all remaining articles. Any discrepancies in the results between the reviewers were resolved by consensus, and if agreement could not be reached, a third researcher (OJH) was consulted.

**Fig. 1:**
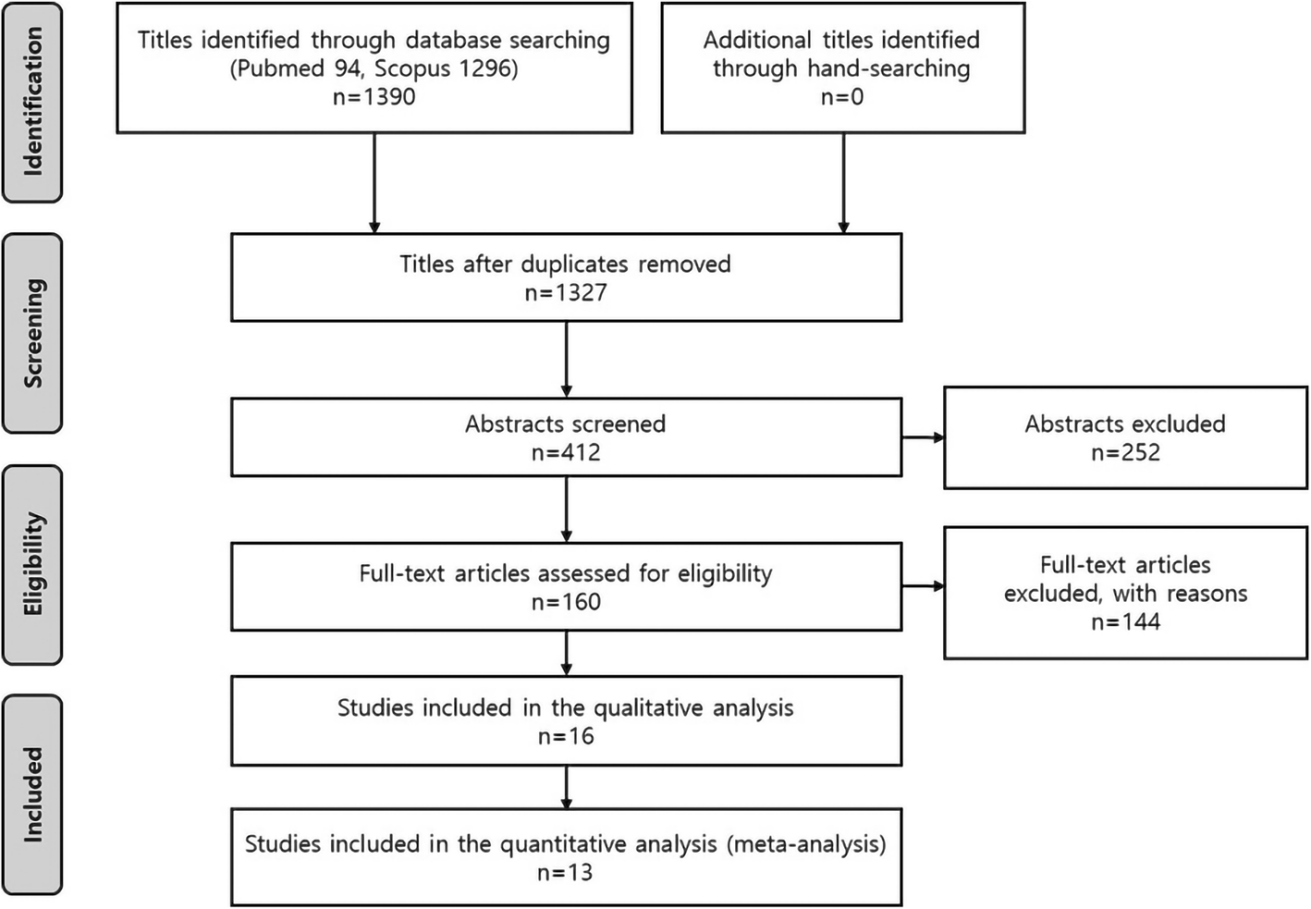
PRISMA flow diagram

The PRISMA flow diagram provides an overview of the study selection process (Fig. 1). Initially, the titles of the identified reports were screened, and duplicates were removed. Abstracts were reviewed if the titles suggested the study was relevant. For studies that appeared relevant or if the abstract was unavailable, a full-text analysis was conducted. Additionally, the references of identified papers and previously published systematic reviews on VSP and TSP in conjunction with orthognathic surgery were cross-checked to ensure no articles were missed.

### Data collection process

Data were extracted by a single reviewer (LYJ) and entered into an Excel spreadsheet (Microsoft, Redmond, WA, USA). The following items were collected: first author, year of publication, journal name, study design, number of patients, mean patient age, types of experimental and control groups, imaging method, type of surgery, type of software used, and outcomes related to surgical accuracy and surgical time. The accuracy was compared using the linear absolute difference between the preoperative plan and the postoperative outcome. Surgical time was included in the meta-analysis only when it reflected the total duration of the surgery. To assess the level of agreement between the evaluators, the kappa statistic was employed using the same criteria as during the study selection phase. Any discrepancies were resolved through discussion between the evaluators; if consensus could not be reached, a third assessor (OJH) was consulted for input.

### Evaluation of the study risk of bias

Due to the diverse study designs of the studies included in this review, two tools were used to assess the risk of bias. The Cochrane Library Risk of Bias (RoB) tool was used for RCTs. RoBANS (Risk of Bias Assessment Tool for Non-randomized Study) tool was used for non-RCTs. There are five types of bias: selection bias, performance bias, detection bias, attrition bias, and reporting bias. The evaluation criteria for the type of bias are somewhat different between RCTs and non-RCTs (Table 2). The risk of bias was classified as either high, low, or uncertain. A risk of bias assessment was undertaken by two review authors (LYJ and KSG).

**Table 2:** The evaluation criteria for the type of bias

Bias type	RoB (for RCTs)	RoBANS (for non-RCTs)
Selection bias (1)	Random sequence generation	Selection of participants
Selection bias (2)	Allocation concealment	Confounding variables
Performance bias	Blinding of participants and personnel	Measurement of exposure
Detection bias	Blinding of outcome assessment	Blinding of outcome assessment
Attrition bias	Incomplete outcome data	Incomplete outcome data
Reporting bias	Selective reporting	Selective reporting
Study designs covered by the tool	Randomized controlled trial	Nonrandomized trials Cohort study Case–control study Before and after study

Selection bias (1) was assessed as random sequence generation for randomized controlled trials (RCTs) and as selection of participants for non-randomized controlled trials (non-RCTs). In both cases, the focus is on the process of selecting study participants

Selection bias (2) was assessed as allocation concealment for randomized controlled trials (RCTs) and as confounding variables for non-randomized controlled trials (non-RCTs). In both cases, there is an emphasis on minimizing external factors that could impact the study outcomes

### Statistical analysis

The absolute mean linear difference values comparing the planned position and the postoperative position were extracted. While angular discrepancy values were included in the qualitative analysis, they were not incorporated into the meta-analysis (quantitative analysis). For absolute mean linear difference values reported as total in 3D, the data were used as is. However, when *x*,*y* values or *x*,*y*,*z* values, along with multiple reference points, were provided, the following formulas were employed to consolidate the data into a single value for each study [11].

When there were *x*, *y* values or *x*, *y*, *z* values, the mean can be calculated using the following formula:

$${\mu }_{D}\approx \sqrt{{\mu }_{x}^{2}+{\mu }_{y}^{2}}$$

$${\mu }_{D}\approx \sqrt{{\mu }_{x}^{2}+{\mu }_{y}^{2}+{\mu }_{z}^{2}}$$

When there were *x*, *y* values or *x*, *y*, *z* values, the standard deviation was obtained using the variance propagation formula.

$${\sigma }_{D}\approx \sqrt{(\frac{{\mu }_{x}}{{\mu }_{D}}{)}^{2}{\sigma }_{x}^{2}+(\frac{{\mu }_{y}}{{\mu }_{D}}{)}^{2}{\sigma }_{y}^{2}}$$

$${\sigma }_{D}\approx \sqrt{\frac{{\sigma }_{x}^{2}.{\mu }_{x}^{2}+{\sigma }_{y}^{2}.{\mu }_{y}^{2}+{\sigma }_{z}^{2}.{\mu }_{z}^{2}}{{\mu }_{D}^{2}}}$$

In the presence of multiple reference points, the average value was obtained using the arithmetic mean formula:

$$\mu =\frac{1}{n}\left({x}_{1}+{x}_{2}+\dots +{x}_{n}\right)$$

In the presence of multiple reference points, the standard deviation was calculated using the pooled standard deviation.

$${S}_{\text{pooled}}=\sqrt{\frac{\left({n}_{1}-1\right){S}_{1}^{2}+\left({n}_{2}-1\right){S}_{2}^{2}+\dots +\left({n}_{k}-1\right){S}_{k}^{2}}{{n}_{1}+{n}_{2}+\dots +{n}_{k}-k}}$$

In cases where *x*, *y* values or *x*, *y*, *z* values were provided from multiple reference points, the three-dimensional values were calculated, considering the presence of these multiple reference points to determine the final value.

When sample size, median, range, and/or interquartile range or minimum and maximum values were available, the sample mean and standard deviation were estimated and calculated accordingly [11]. Data were analyzed using RevMan software (The Cochrane Collaboration, Copenhagen, Denmark), employing a random-effects model with inverse variance weighting to estimate the mean differences, along with 95% confidence intervals (CIs). Heterogeneity among the studies was assessed using statistical measures including Tau-squared (τ2), chi-squared (*χ*2) tests, degrees of freedom (df), and the *I*^2 statistic.^ To further explore the meta-analysis results, we conducted a sensitivity analysis by sequentially removing each study to evaluate its impact on the overall findings. This approach allowed us to determine the robustness of the results and assess the potential influence of any single study on the overall effect estimates.

## Results

### Study selection

The search results are summarized in Fig. 1. Through database searching, a total of 1390 articles were identified, with no additional articles found through hand-searching. After removing duplicates, 1327 articles remained. A title screening process reduced this number to 412. Following the review of abstracts, 160 articles were selected for further evaluation. A full-text review of these 160 articles resulted in 16 studies remaining. Ultimately, 13 studies were included in the meta-analysis.

### Characteristics of the included studies

Among the 13 selected studies, 10 reported surgical accuracy (absolute mean linear difference), while 4 addressed operative time (Table 3). One study provided data on both surgical accuracy and operative time. Notably, among the 10 studies that focused on accuracy, 3 compared VSP method, including custom surgical guides, with conventional techniques. The study designs comprised 6 retrospective studies and 7 randomized controlled trials (RCTs).

**Table 3:** Study characteristics

No	Author (publication year)	Design study	Experimental group	Control group	Total patient			Age		Extracted data	Imaging	Type of surgery	Type of software
					E	C	T	E	C				
1	Ritto et al. (2018) [12]	Retrospective	VSP (CAD/CAM printing of splints)	CMS (dental casts were mounted into an articulator, conventional intermediate splint)	15	15	30	ND	ND	Accuracy	CT	Bimaxillary orthognathic surgery	Dolphin Imaging Software (Dolphin Imaging and Management Solutions, Chatsworth, CA, USA)
2	Quast et al. (2021)[13]	Retrospective	VSP (CAD/CAM printing of splints)	CSP (dental casts were mounted into an articulator, conventional intermediate splint)	26	26	52	25.9 (SD 7.1)	22.9 (SD 4.8)	Accuracy	CBCT	LFI or Bimaxillary osteotomy	ProPlan CMF (Materialise, Leuven, Belgium)
3	Schneider et al. (2018)[14]	RCT	VSP (CAD/CAM printing of splints, pre-bent osteosynthesis plates)	CSP (analysis of the lateral radiograph, orthopantomogram/CBCT, facial arch, and articulated plaster models, production of intermediate and definitive splints)	9	12	21	31.1 (median: 32.6, minimum: 23, maximum: 52.1)		Surgical time	–	Bimaxillary orthognathic surgery	Dolphin 3D Imaging (Dolphin Imaging 11.9 Premium and Management solutions, Chatsworth, CA, USA)
4	Kwon et al. (2014) [15]	Retrospective	VMS (virtual model surgery and 3D printed intermediate splint)	AMS (conventional articulator model surgery and acrylic resin intermediate splint)	19	23	42	21.9 (SD 3.0)	23.1 (SD 5.2)	Accuracy	2D lateral and PA cephalograms with scanned dental cast	One-piece Le Fort I osteotomy and sagittal split ramus osteotomy	3Txer program (3Txer ver. 2.5; Orapix, Seoul, Korea)
5	Hemelen et al. (2015) [16]	RCT	3D computer-aided planning approach (3D virtual planning, intermediate and final surgical splints were virtually constructed)	Traditional 2D planning technique (lateral and frontal cephalogram analysis, surgical splints were fabricated by a dental laboratory)	31	35	66	19.78		Accuracy	CBCT	Bimaxillary osteotomy(46 patients), Bilateral sagittal split osteotomy (17 patients), LFI osteotomy(3 patients)	Maxilim software (Medicim NV, Mechelen, Belgium)
6	Hanafy et al. (2020) [17]	RCT	CAD/CAM group (CAD/CAM generated surgical guides and patient-specific titanium plates for maxillary positioning)	Classic wafer group (inter-occlusal wafers fabricated on a semi-adjustable articulator for maxillary positioning)	9	9	18	21.22		Accuracy	CBCT	Orthognathic surgery	Mimics 19.0 (Materialise NV, Leuven, Belgium)
7	Xu et al. (2020)[18]	RCT	VSP (virtual surgical planning and splint printing)	AMS (articulator model surgery and conventional splint fabrication)	15	15	30	25	25	Accuracy	CBCT	Double jaw surgery (LFI and BSSRO)	Geomagic Studio (v2012, Materialse) Freeform (v.2013, SensAble)
8	Chen et al. (2021) [19]	RCT	DT group (digital templates) (printed digital cutting and repositioning templates and final splint)	CROS group (conventional resin intermediate and final splints)	20	20	40	24(SD 4)	23(SD 3)	Accuracy	CT	Bimaxillary surgery	Mimics 19.0 software (Materialse, Leuven, Belgium)—to reconstruct a 3D digital model 3-Matic 11.0 software—for an intermediate digital splint
9	Lin et al. (2020) [20]	Retrospective cohort study	VSP (3D-printed splints)	TSP (conventional splints)	19	10	29	17.66(SD 0.93)	19.35(SD 2.84)	Surgical time	–	Both maxillary and mandibular osteotomies	Materialise NV, Leuven, Belgium
10	Schwartz et al. (2014) [21]	Retrospective study	Computer-aided surgical simulation (CASS)—computer-aided surgical simulation, CAD/CAM splint	Traditional methods (cephalometric analysis, model surgery, splint construction)	30	30	60	28.3 (SD 10.547, range 16–54)	25.6 (SD 9.092, range 16–49)	Surgical time	–	Maxillary and mandibular osteotomies and adjunctive procedures	ND
11	Kraeima et al. (2020) [22]	RCT	Patient-specific osteosynthesis (3D printed drilling/osteotomy guide, CAD/CAM surgical splint)	Conventional osteosynthesis and splint-based positioning	27	31	58	27.6 (SD 10)	29.5 (SD 9)	Accuracy	CBCT	LFI	Maxilim v2.3 (Maxilim; Medicim NV, Mechelen, Belgium)
12	Bengtsson et al. (2017) [23]	RCT	3D prediction technique	2D prediction technique	28	29	57	21.1	20.5	Accuracy	CT	LFI, Segmented Le Fort I maxillary osteotomy, Bilateral sagittal split mandibular osteotomy, vertical ramus mandibular osteotomy, and genioplasty	Simplant PRO 12.02 OMS (Materialise Corp., Leuven, Belgium)
13	Yamaguchi et al. (2020) [24]	Retrospective study	CAD/CAM group (CAD/CAM intermediate wafers)	Conventional group (conventional model surgery, conventional intermediate wafer)	14	10	24	20.4 (range 16–28 years)	25.6 (range 16–44 years)	Accuracy Surgical time	CT	Bimaxillary surgery(Le Fort I osteotomy, bilateral sagittal split osteotomy)	ProPlan CMF (Materialise, Leuven, Belgium)

### Exclusion of studies

Three papers met the inclusion criteria but were excluded from the meta-analysis for the following reasons. Zinser et al. [25] compared linear measurements between preoperative simulated virtual positions and actual postoperative results, but while the mean value and *p*-value were reported, the standard deviation could not be calculated from the available data. In Barone et al. [26], accuracy was reported by comparing traditional surgical planning and digital surgical planning, but the only outcome indicator was angular discrepancy. Bengtsson et al. [27] reported the difference between planning and result, but it was not expressed as an absolute mean value.

A sensitivity analysis was conducted by sequentially removing one study at a time to observe its impact on the overall effect estimate. This approach helped evaluate the robustness of the findings and determine the influence of individual studies on the overall effect size. While the results were generally stable, the exclusion of Kraeima et al. [22] led to a significant increase in the overall effect Z-score from 3.53 to 10.57, indicating that the overall findings are sensitive to the presence of this study. Conversely, in the meta-analysis comparing VSP to CSP, the exclusion of the studies by Chen et al. [19], Hemelen et al. [16], and Kraeima et al. [22] resulted in a decrease in the overall Z-score, suggesting that these studies contributed positively to the effect estimate. Despite these variations, the overall conclusions remained consistent with the pooled results from the included studies.

### Risk of bias assessment

**Fig. 2:**
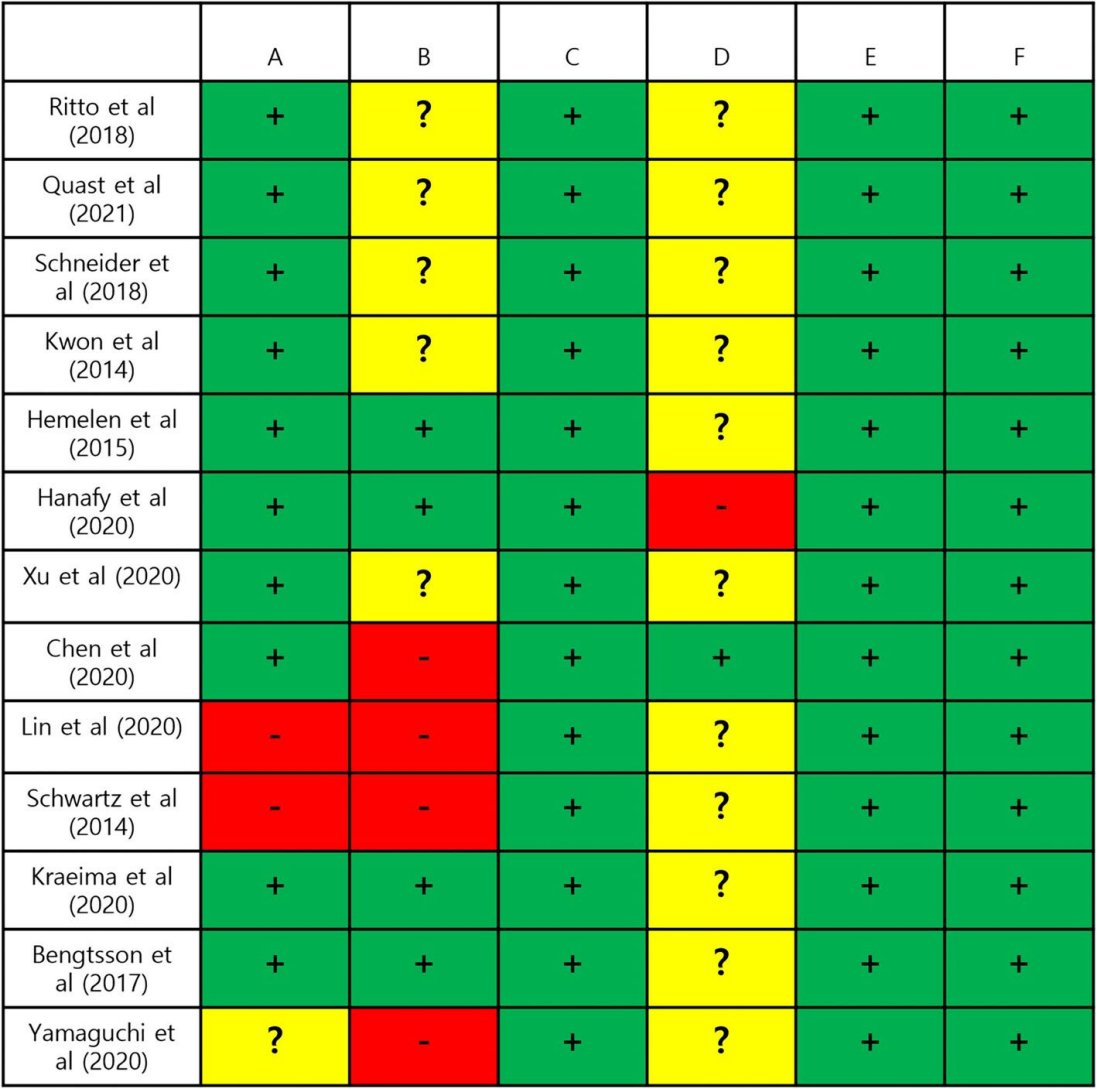
Risk of Bias Assessment Across Included Studies. This figure displays the risk of bias assessment for each included study across various domains. Green ( +) indicates a low risk of bias, yellow (?) denotes an unclear risk of bias, and red ( −) represents a high risk of bias. The domains assessed include **A** random sequence generation, **B** allocation concealment, **C** blinding of participants and personnel, **D** blinding of outcome assessment, **E** incomplete outcome data, **F** selective reporting, and other biases. This visual summary provides an overview of the methodological quality and potential sources of bias within each study

For comparison of VSP to the classic method in terms of the accuracy of orthognathic surgery, 10 studies were (Fig. 2). The risk of bias was assessed using the Cochrane RoB Tool for RCTs and RoBANS (Risk of Bias Assessment tool for Non-randomized Studies) for non-RCTs. Overall, the studies demonstrated varying levels of methodological quality. For random sequence generation and selection of participants, 10 out of 13 studies were judged as having a low risk of bias.

Regarding allocation concealment and confounding variables, the studies showed more variability. Four studies were assessed as having a low risk of bias, while five were classified as unclear risk due to insufficient information. Four studies were judged to have a high risk of bias, indicating potential flaws in allocation concealment and the handling of confounding variables.

In the domain of performance bias, all studies were judged to have a low risk of bias. For the outcomes related to surgical accuracy and operative time, it was determined that performance bias was not a concern, even if participants or personnel were aware of the intervention.

### Accuracy of VSP

**Fig. 3:**
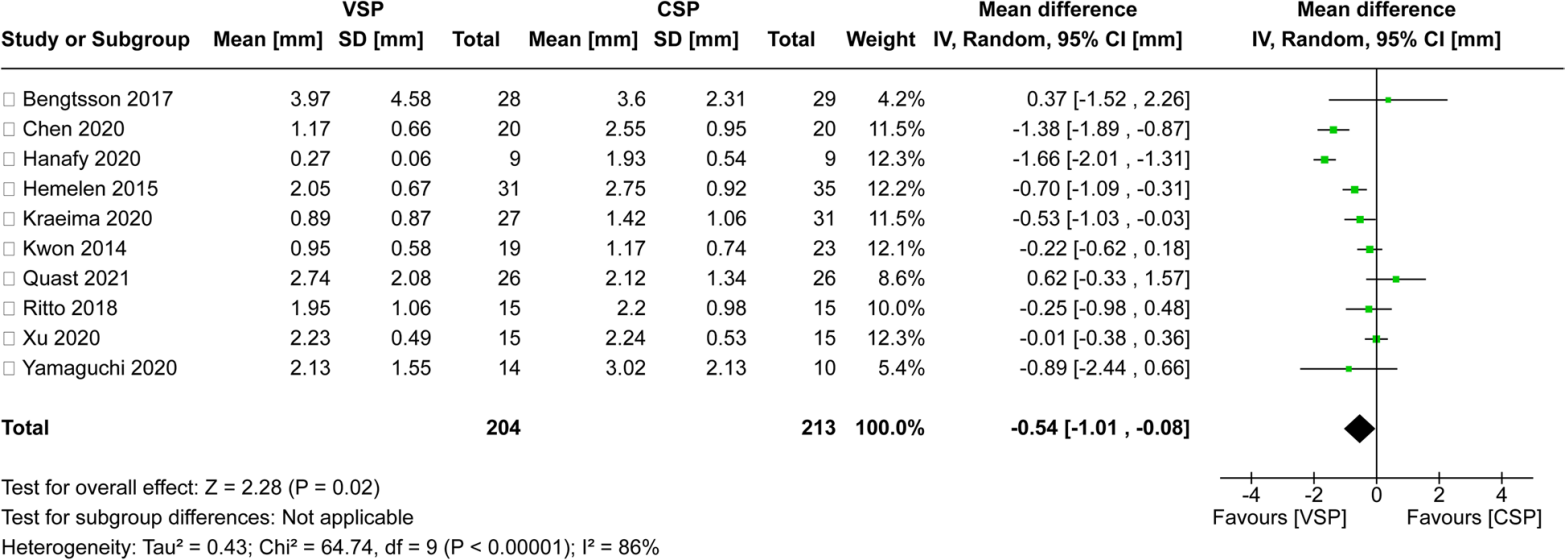
Forest Plot for comparison of accuracy between VSP and CSP. This forest plot displays the mean differences (with 95% confidence intervals) between the VSP and CSP groups across various studies on the accuracy of surgical planning methods. The summary effect (black diamond) indicates an overall mean difference of − 0.54 mm, favoring the VSP approach (*P* = 0.02)

For comparison of VSP to the classic method in terms of the accuracy of orthognathic surgery, 10 studies were selected (Fig. 3). When the heterogeneity of the articles was checked, it showed a significantly high level of heterogeneity (Tau^2^ = 0.43; chi^2^ = 64.74, df = 9 (*P* < 0.00001); *I*^2^ = 86%). Accordingly, a meta-analysis was conducted using a random-effects model. The results indicated that VSP demonstrated a 0.54-mm lesser difference between the planned and actual jaw bone positions compared to the classic method (95% CI − 1.01 mm to − 0.08 mm), with a statistically significant difference between groups (*P* = 0.02).

**Fig. 4:**
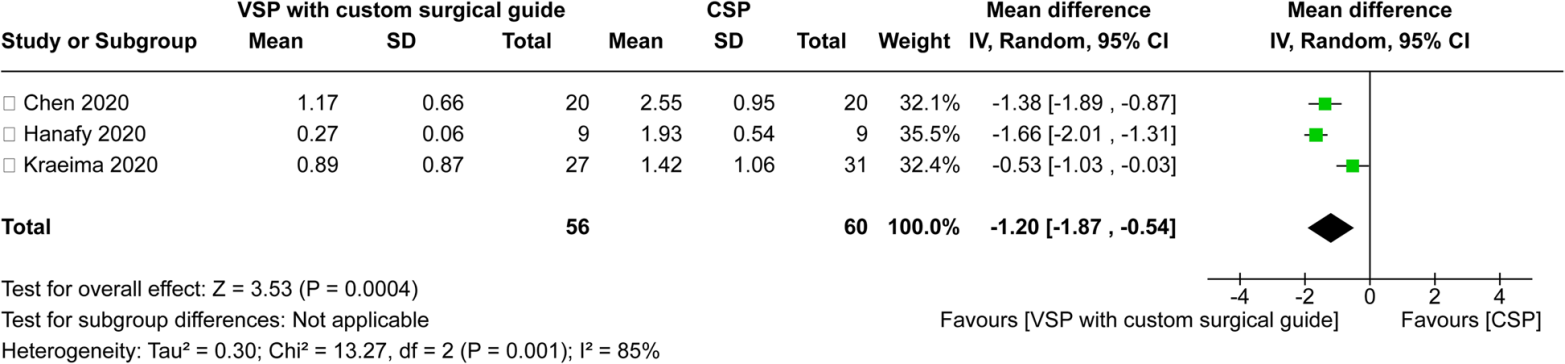
Forest Plot for comparison of accuracy between VSP with surgical guide and CSP. This forest plot displays the mean differences (with 95% confidence intervals) between the VSP with surgical guide and CSP groups across various studies on the accuracy of surgical methods. The summary effect (black diamond) indicates an overall mean difference of − 1.20 mm, favoring the VSP with surgical guide approach (*P* = 0.0004)

Building upon this, when comparing VSP—including the custom surgical guide—to the classic method, 3 out of the 10 studies were selected (Fig. 4). The heterogeneity analysis revealed a high level of variability (Tau^2^ = 0.30; chi^2^ = 13.27, df = 2 (*P* = 0.001); *I*^2^ = 85%). A meta-analysis was similarly performed using a random-effects model. Notably, the VSP with the custom surgical guide group showed a 1.20-mm lesser difference between the planned and actual jaw bone positions compared to the classic method (95% CI − 1.87 mm to − 0.54 mm). This difference was statistically significant (*P* = 0.0004), indicating even more pronounced results when utilizing the custom surgical guide.

### Operation time of VSP

**Fig. 5:**
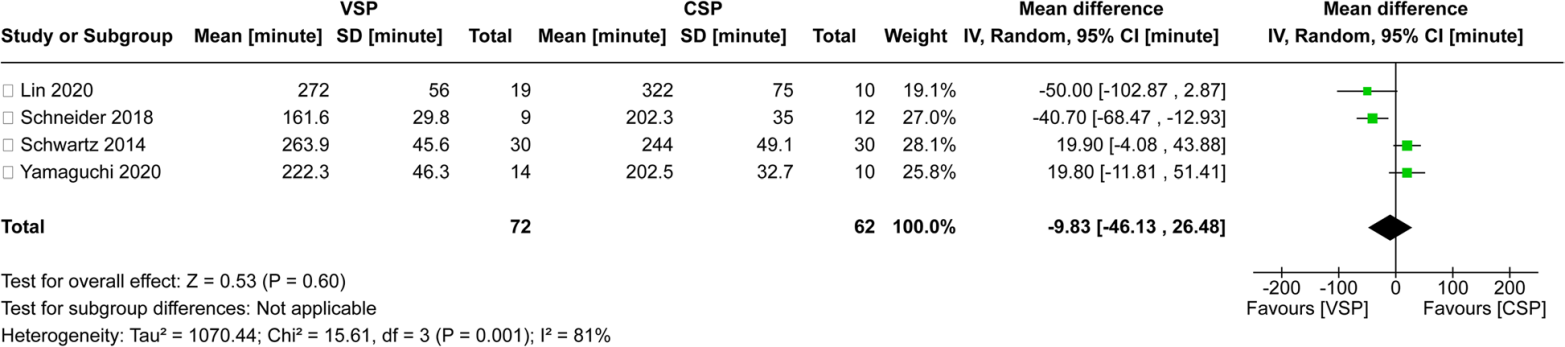
Forest Plot for comparison of operation time between VSP and CSP. This forest plot displays the mean differences (with 95% confidence intervals) between the VSP and CSP groups across various studies on the operation time of methods. The summary effect (black diamond) indicates an overall mean difference of 9.83 min, favoring the VSP approach. However, the difference between groups was not significant (*P* > 0.05)

To compare the operation time of VSP with the classic method, 4 studies were selected for analysis (Fig. 5). A heterogeneity assessment indicated a significantly high level of variability among the articles (Tau^2^ = 1070.44; chi^2^ = 15.61, df = 3 (*P* = 0.001); *I*^2^ = 81%). Consequently, a meta-analysis was conducted using a random-effects model. The results revealed that VSP saved an average of 9.83 min compared to the classic method (95% CI − 46.13 min to 26.48 min). However, the difference between the two groups was statistically insignificant (*P* = 0.60).

## Discussion

Accurate pre-surgical planning is fundamental to successful orthognathic surgery, as it establishes the foundation for precise surgical interventions aimed at correcting complex maxillofacial deformities [5, 28]. The digital diagnosis phase utilizes advanced imaging techniques, with CBCT serving as a cornerstone [29]. CBCT provides highly detailed, three-dimensional images of the patient’s craniofacial structures, which are crucial for identifying specific anatomical features and planning surgical interventions with precision [30]. The detailed data obtained from CBCT scans are then integrated into specialized orthognathic surgery software [6, 7], allowing for the construction of a 3D virtual patient model—a pivotal step in surgical planning [31]. This process creates an accurate representation of the patient's bone anatomy, intraoral tissues, and facial soft tissues [5]. The precision and clarity offered by this virtual model are vital for planning complex surgical procedures with greater confidence and accuracy [6, 10]. The evolution of VSP now encompasses various methodologies [32], including the creation of intermediate occlusal wafers via CAD/CAM technology, the production of osteotomy guides, and the development of patient-specific implants or customized osteosynthesis plates. In this study, VSP demonstrated better accuracy compared to CSP (Fig. 3). Furthermore, when VSP was combined with custom surgical guides, the accuracy was enhanced even more (Fig. 4).

### Accuracy of VSP

In this meta-analysis, a search for published comparative analyses was conducted. Unfortunately, there are few comparative studies on this topic. Only 10 articles were found and some of them were retrospective studies. The studies by Habib et al. [33] and Imai et al. [34] offer insights into the accuracy of 3D printing and computer-aided design/computer-aided manufacturing (CAD/CAM) technologies in orthognathic surgery. Alqussair et al. [35] highlight the precision in maxilla positioning for skeletal class II malocclusion with notable mean absolute deviations. Their findings indicate mean absolute deviations of 0.98 mm in the sagittal coordinate, 0.67 mm in the vertical, and 0.62 mm in the transverse coordinate, showcasing the high degree of surgical accuracy and clinical relevance of this technique in orthognathic surgery [35].

In a randomized controlled trial comparing CAD/CAM cutting and drilling guides with CAD/CAM splints, no significant difference was found in the correction of skeletal class II malocclusion [36]. A recent systematic review indicates that in computer-aided planning for orthognathic surgery, the translational accuracy is generally less than 1.2 mm for the maxilla and less than 1.1 mm for the mandible [37]. The accuracy level in the maxilla aligns with previous findings [38] and is comparable to the accuracy achieved by classical methods, which is less than 1.3 mm [1]. This suggests a consistency in the precision of these orthognathic surgery techniques over time and no significant improvement by using CAD/CAM splint.

Chen et al. [39] evaluate the accuracy of computer-aided intraoperative navigation in bimaxillary surgery, underscoring its superiority over conventional methods. This is complemented by research showing significant differences in the alignment of the lower interincisal point, mandibular sagittal plane, and dental midlines alignment when comparing surgical navigation with CAD/CAM splints to traditional methods, with digital planning proving more accurate [40]. Zinser et al. [1] noted that the greatest accuracy in transferring maxillary planning was observed with CAD/CAM splints, where the deviation from the surgical plan to the actual outcome was less than 0.23 mm. This precision was comparatively higher than that achieved with surgical “waferless” navigation, which had a deviation of less than 0.61 mm, and traditional intermaxillary occlusal splints, with a deviation of less than 1.1 mm. Ha et al. [41, 42] discuss the effectiveness of 3D virtual planning in facial asymmetry surgeries and skeletal class III patients, emphasizing the importance of advanced technologies. The study found that the total error rate for maxillary repositioning in orthognathic surgery was 0.62 mm, as measured by comparing the postoperative CBCT scans with the preoperatively planned 3D skull models [43]. The studies by Hsu et al. [44] and Ritto et al. [12] focus on specific surgical areas, attributing improved accuracy to surgical splints and surgeons’ experience. Koyachi et al. [45] used comparative statistics such as the Mann–Whitney *U* test and Kruskal–Wallis test and concluded that there were no statistically significant differences between any of the points on any of the axes between plan and actual surgical outcome. Similarly, de Britto Teixeira et al. [46] used comparative statistics such as paired *t* test and had similar conclusions.

Ho et al. [47] compare traditional hybrid and full 3D digital planning models, showing significant improvements in post-surgery outcomes. De Riu et al. [48] evaluate an in-house protocol for computer-assisted surgery using resin-printed guides, demonstrating the reliability and accuracy of in-house 3D printing technologies. Collectively, these studies paint a comprehensive picture of the advancements in orthognathic surgical techniques, showcasing a paradigm shift towards enhanced precision and predictability. The article by Denadai et al. [49] discusses a 3D computer-assisted single-splint two-jaw orthognathic surgery technique for cleft skeleton-facial reconstruction. This approach is tailored to the specific needs of skeletally mature patients with cleft lip and palate, who often present with complex dentoskeletal and soft tissue abnormalities. In the context of cleft patients undergoing orthognathic surgery, VSP exhibits a discrepancy level of 2.75 mm between the planned and actual outcomes in the maxilla [50]. This discrepancy is notably larger compared to outcomes observed in non-cleft patients, indicating a potential challenge in achieving the same level of accuracy in surgical planning for cleft-affected individuals. The accuracy of VSP in orthognathic surgery is also influenced by the surgeon's experience [51]. The effectiveness of VSP in achieving the desired surgical outcomes can vary depending on the surgeon's skill and familiarity with the technology and techniques involved.

### Operation time of VSP

The forest plot showed that VSP showed less operation time compared to CSP, but the difference between the groups was insignificant. Karwowska et al. [52] emphasize that surgeons primarily use VSP to enhance the accuracy, efficiency, and ease of preoperative planning. This approach reduces overall surgical time, with most surgeons perceiving VSP to be as fast as or faster than traditional methods [52]. The data generated through these processes ensures that each guide is customized to the patient's specific anatomical requirements, significantly reducing operative time by providing clear and precise directions for the surgical procedure [53]. However, Hu et al. [54] noted that while drilling guides and pre-bent plates can be advantageous compared to occlusal splints, the production of these guides and templates is time-consuming and costly. According to a recent meta-analysis, operation time was not significantly reduced by VSP [55] and it was in accord with our study.

### Accuracy and operation time of VSP including custom surgical guide

Three studies compared the accuracy of VSP with custom surgical guides to CSP and were included in the meta-analysis (Fig. 4). Hanafy et al. [17] concluded that the computer-assisted workflow for orthognathic surgery offers a more precise transfer of plans compared to traditional occlusal wafers [17]. They reported average surgery times—from maxillary incision to fixation—of 49 min for the computer-aided surgery group and approximately 72 min for the classic inter-occlusal wafers group. However, their intraoperative time data could not be included in the meta-analysis due to the inability to calculate the standard deviation. Chen et al. [22] found that the digital templates group exhibited the smallest deviation between the actual and planned positions of the maxilla compared to the conventional resin occlusal splint group. Although they reported surgical times, they provided data only for specific stages of the surgery rather than the total duration, leading to their exclusion from the meta-analysis for surgical time. The digital templates group had an average time of 50.0 ± 18.0 min, while the conventional resin occlusal splint group reported 41.7 ± 13.1 min. Kraeima et al. [19] concluded that the use of 3D virtual surgical planning, along with drilling and cutting guides and patient-specific osteosynthesis, enhances the accuracy of maxillary translations in orthognathic surgery.

Two studies [56, 57] compared surgical accuracy between groups utilizing cutting guides and custom osteosynthesis plates versus those using conventional plates. Both studies employed computer-aided surgical simulation for all groups, which precluded their inclusion in the meta-analysis comparing VSP with CSP. In the first study [56], the mean difference in accuracy was reported as 0.485 ± 0.280 mm for the group using 3D-printed patient-specific plates and osteotomy guides, compared to 1.213 ± 0.716 mm for the group utilizing conventional plates, suggesting improved accuracy with custom plates. Conversely, the second study [57] investigated whether customized CAD/CAM cutting guides and custom osteosynthesis plates improve the accuracy of proximal segment positioning during bilateral sagittal split osteotomy and concluded that customized mandibular fixation plates do not necessarily enhance the postoperative accuracy of the proximal segments. Therefore, when VSP is used, the addition of custom plates may or may not improve surgical accuracy, indicating that their benefit is not consistent across studies.

### Limitation of this study

Different studies used various landmarks to evaluate the accuracy of their techniques, resulting in variations in the measured values. Even if some landmarks remained in their planned positions, others might not, particularly if rotational movements occurred around a fixed landmark. Therefore, in this study, the changes from all measured landmarks were averaged. For any detected movement along each axis, the actual linear movement was calculated. While some studies employed angular measurements for accuracy evaluation, this study did not consider angular measurements because different 3-dimensional positions can yield the same angular value.

For retrospective studies, there is a potential for a higher level of selection bias. Because the data has already been collected for other purposes, the selection of participants is not randomized or controlled, leading to potential selection bias. In addition, the planned positions in this study were primarily evaluated in two dimensions. If the comparison between planned and actual positions is conducted in a 2-dimensional space, it means that only two axes (such as *X* and *Y*) are being considered for measurement and analysis. This approach inherently neglects the third axis (*Z*-axis) in a 3-dimensional space. Additionally, many studies did not clearly state whether the accuracy measurements were performed in a blinded manner. Since researchers often prefer newer techniques, if the accuracy assessments were not conducted blindly, the results could be subject to bias.

## Conclusion

This study evaluated the accuracy and operation time of VSP compared to the CSP for orthognathic surgery through a meta-analysis of 13 studies. The findings suggest that VSP especially when including custom surgical guides is more accurate, showing a statistically significant reduction in the difference between planned and actual jaw bone positions compared to CSP. However, while the VSP approach demonstrated a potential reduction in operation time compared to CSP, this difference was not statistically significant. These results indicate that VSP-based custom surgical guides improve accuracy in orthognathic surgery, but further research is needed to confirm their impact on reducing operation time, considering the high heterogeneity among the included studies.

## Data Availability

No datasets were generated or analysed during the current study.

## Abbreviations

CAD/CAM: Computer-aided design/computer-aided manufacturing
CBCT: Cone beam computed tomography
FDM: Fused deposition modeling
PSP: Patient-specific plates
SLA: Stereolithography
SLS: Selective laser sintering
VSP: Virtual surgical planning
